# A deep intronic splice variant advises reexamination of presumably dominant *SPG7* Cases

**DOI:** 10.1002/acn3.50967

**Published:** 2019-12-18

**Authors:** Edgard Verdura, Agatha Schlüter, Gorka Fernández‐Eulate, Raquel Ramos‐Martín, Miren Zulaica, Laura Planas‐Serra, Montserrat Ruiz, Stéphane Fourcade, Carlos Casasnovas, Adolfo López de Munain, Aurora Pujol

**Affiliations:** ^1^ Neurometabolic Diseases Laboratory Bellvitge Biomedical Research Institute (IDIBELL) L'Hospitalet de Llobregat Barcelona Catalonia Spain; ^2^ Centre for Biomedical Research on Rare Diseases (CIBERER) Instituto de Salud Carlos III Madrid Spain; ^3^ Biodonostia Neurosciences Area Neuromuscular diseases Laboratory San Sebastian Basque country Spain; ^4^ CIBERNED Instituto de Salud Carlos III Ministry of Science, Innovation and Universities Madrid Spain; ^5^ Department of Neurology Hospital Universitario Donostia San Sebastian Basque country Spain; ^6^ Neuromuscular Unit Neurology Department Hospital Universitari de Bellvitge, L'Hospitalet de Llobregat Barcelona Catalonia Spain; ^7^ Department of Neurosciences Faculty of Medicine and Dentistry UPV‐EHU San Sebastian Basque country Spain; ^8^ Catalan Institution of Research and Advanced Studies (ICREA) Barcelona Catalonia Spain

## Abstract

**Objective:**

To identify causative mutations in a patient affected by ataxia and spastic paraplegia.

**Methods:**

Whole‐exome sequencing (WES) and whole‐genome sequencing (WGS) were performed using patient's DNA sample. RT‐PCR and cDNA Sanger sequencing were performed on RNA extracted from patient's fibroblasts, as well as western blot.

**Results:**

A novel missense variant in *SPG7* (c.2195T> C; p.Leu732Pro) was first found by whole‐exome sequencing (WES), while the second, also unreported, deep intronic variant (c.286 + 853A>G) was identified by whole‐genome sequencing (WGS). RT‐PCR confirmed the *in silico* predictions showing that this variant activated a cryptic splice site, inducing the inclusion of a pseudoexon into the mRNA sequence, which encoded a premature stop codon. Western blot showed decreased SPG7 levels in patient's fibroblasts.

**Interpretation:**

Identification of a deep intronic variant in *SPG7*, which could only have been detected by performing WGS, led to a diagnosis in this HSP patient. This case challenges the notion of an autosomal dominant inheritance for *SPG7*, and illustrates the importance of performing WGS subsequently or alternatively to WES to find additional mutations, especially in patients carrying one variant in a gene causing a predominantly autosomal recessive disease.

## Introduction

Hereditary spastic paraplegias (HSPs) are a group of neurodegenerative diseases characterized by progressive loss of upper motor neuron function. The primary pathophysiological feature of the disease is a “dying back type” axonal neuropathy that primarily affects upper motor neurons of the pyramidal tract.^1^, ^2^ Collectively, more than 80 genes and at least 76 linkage loci have been associated with HSP.^3^, ^4^ Among all of these genes, mutations in *SPAST*, *ATL1*, *SPG7*, and *SPG11* remain the most prevalent causes of HSP. Novel sequencing strategies such as whole‐exome sequencing (WES) have accelerated the identification of causative variants associated with HSP, including the identification of novel gene–disease associations,^5^ although the diagnostic rate reaches only ~ 50% in carefully selected cohorts of patients.^6^, ^7^ Whole‐genome sequencing (WGS) can improve the detection of variants, as it provides uniform coverage of exonic regions (especially important to screen regions that are not efficiently probed by WES), the possibility of detecting deep intronic/intergenic variants, and improved calling of structural variants.^8^ Although WGS has a higher economic cost compared to WES and the interpretation of non‐coding variants remains a challenge, it has already been used successfully to find pathogenic variants in HSP patients and other related neurological conditions (Table S1). In this report, we describe a patient in which a missense heterozygous variant in *SPG7* was first found by WES. To find additional mutations, we performed WGS, which identified a second heterozygous deep intronic variant in *SPG7* that could not have been detected by WES alone. After functional analysis, we concluded that this second deep intronic variant in *SPG7* is responsible for this patient's disease.

## Methods

### Patients and samples

The proband was recruited and examined at the Neurology department of Donostia University Hospital. Detailed neurological examinations were performed on all members of the family. Blood samples and skin‐derived fibroblast cell lines were obtained using standard methods. Informed consent was obtained from all participants in this study. The research project was approved by the Clinical Research Ethics Committee for Research Ethics Committee of the Bellvitge University Hospital (PR076/14).

### Whole‐Exome (WES) and Whole‐Genome Sequencing (WGS)

Genomic DNA was extracted from peripheral blood using standard methods. WES was performed on patient's DNA sample using the SureSelect XT Human All Exon V5 50 Mb kit (Agilent) for DNA capture. Exome and Genome DNA sequencing was performed using the HiSeq 2000 Platform (Illumina) at CNAG (Centre Nacional d'Anàlisi Genòmica, Barcelona). Variants were filtered using RD‐Cat platform (https://rdcat.cnag.crg.eu/) and an in‐house pipeline based on GATK best practice guidelines and prioritization based on accurate patient characterization with HPO terms (Human Phenotype Ontology), interaction networks at physical and functional levels and variant intolerance scores generated by the ExAC and gnomAD consortia. We prioritized variants in HSP genes that had a frequency lower than 0.01 in ExAC (Exome Aggregation Consortium), 1000 genomes, ESP (NHLBI Exome Sequencing Project), and gnomAD control databases. *In silico* predictors were used to assess the effect of variants on splicing (Human Splicing Finder, MaxEntScan, NNSplice, NetGene2, FSplice, and SpliceAI^9^). Furthermore, we screened a curated list of HSP (79), spinocerebellar ataxia (49), and spastic ataxia genes (7). Candidate variants were validated and tested for cosegregation in all family members by Sanger sequencing.

### RNA splicing analysis

Fibroblast cell lines from the proband, a healthy brother, and an unrelated control were cultured at 37ºC and 5% CO_2_ in Dulbecco's modified eagle medium (DMEM) supplemented with 10% fetal bovine serum (FBS) and 100U/mL penicillin + 100µl/mL streptomycin. RNA was extracted using the RNeasy Mini Kit (QIAGEN) and cDNA was synthesized using the Superscript IV kit (Life Technologies) following manufacturer's instructions. cDNA was amplified by polymerase chain reaction (PCR) using primers targeting exons 1–4 of *SPG7* gene (forward: 5′‐CGGCTTTCAGGCCAACAT‐3′; reverse: 5′‐CGCTCTCGGTACATCTGGTC‐3′) followed by Sanger sequencing.

### Western blot

For western blotting analyses, fibroblasts were homogenized in RIPA buffer (150 mmol/L NaCl, 1% Nonidet P40, 0.5% sodium deoxycholate, 0.1% SDS, 50 mmol/L Tris, pH 8.0), sonicated for 2 min at 4ºC, centrifuged, mixed with 4X NuPAGE LDS Sample Buffer (Invitrogen) and heated at 70ºC for 10 min. 60 µg of protein samples was subjected to polyacrylamide gel electrophoresis at 120V in NuPAGE MOPS SDS Running Buffer (Invitrogen) supplemented with 5 mmol/L sodium bisulfite (Ref. 243973, Sigma‐Aldrich). Proteins were transferred to nitrocellulose membranes using iBlot 2 Gel Transfer Device (Invitrogen). After blocking in 5% Bovine Serum Albumin (BSA, Sigma‐Aldrich), 0.05% TBS‐Tween (TBS‐T) for 1 h at room temperature, membranes were incubated with primary antibodies overnight at 4°C. Primary antibodies included Anti‐β‐Actin, mouse (dilution 1/8000, ref:A2228, Sigma‐Aldrich); Anti‐OPA1, mouse (dilution 1/1000, ref: 612607, BD Transduction Laboratories); and Anti‐SPG7, mouse (dilution: 1/500, ref: SC‐514393, Santa Cruz Biotechnology). After incubation with secondary antibodies for 1 h at room temperature, proteins were detected with Chemidoc^TM^ Touch Imaging System (BioRad). Bands were quantified with ImageLab (BioRad).

## Results

### Clinical description

The proband (patient II.1) is a 52‐year‐old man in ongoing follow‐up at the neurology outpatient clinic of Donostia University Hospital due to a 15‐year‐long history of altered gait. Spastic paraplegia with gait ataxia was observed at the first examination in 2005. Mild progressive external ophthalmoplegia (PEO) with ptosis and cerebellar dysarthria were also present. This patient is the first of three sons of a non‐consanguineous couple of Spanish origin. His father had suffered from a paraneoplastic anti‐Hu‐positive cerebellar and polyneuropathic ataxia associated with small‐cell lung cancer and recently died at the age of 73 years. There are no other family members suffering from any disease affecting the central or peripheral nervous system.

**Figure 1:**
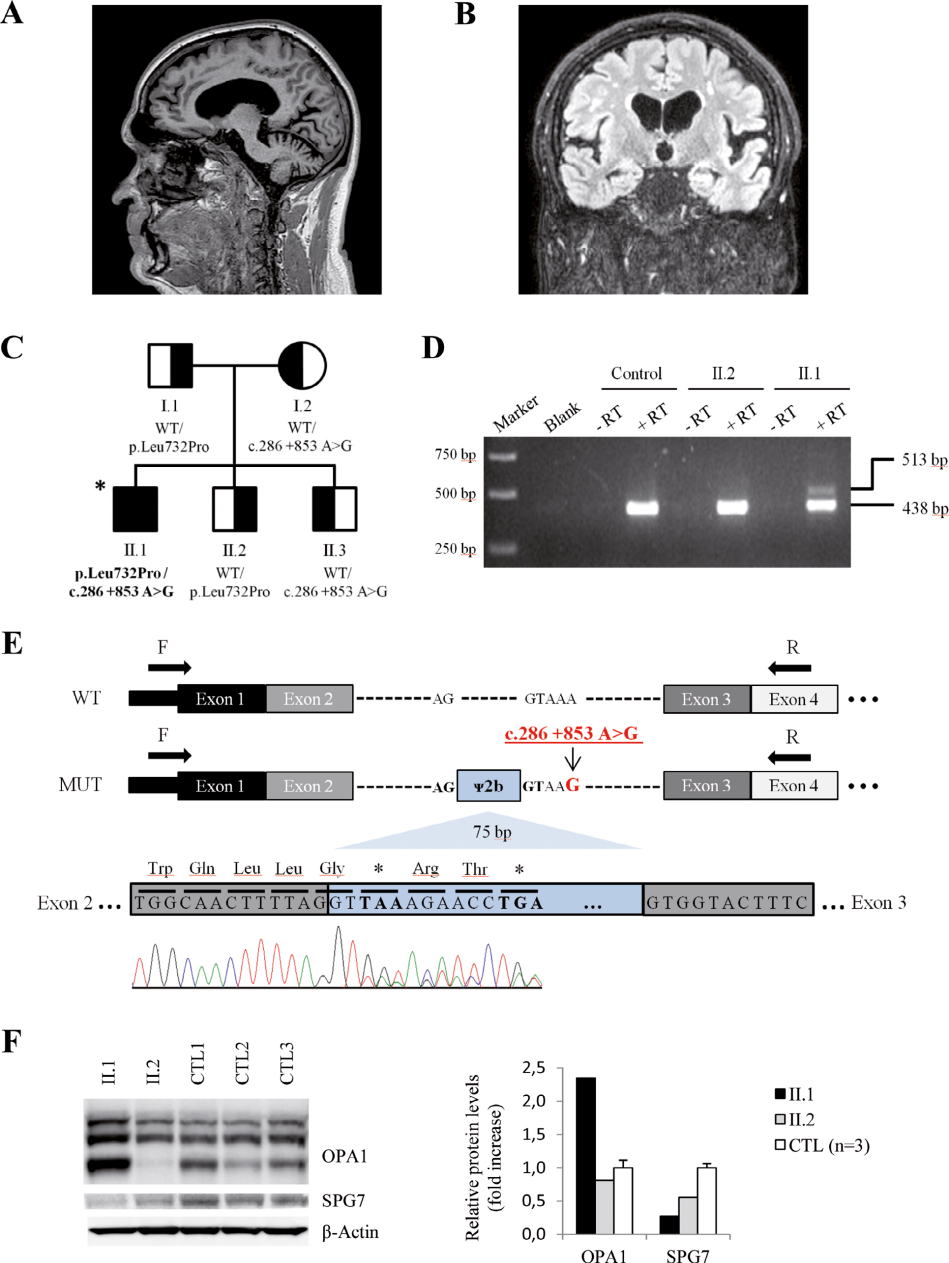
MRI brain images and functional evaluation in fibroblasts from Patient II.1. (A, B) Brain MRI of subject II.1 showing typical cerebellar atrophy of SPG7 disease (sagittal T2w) (A), and bilateral frontotemporal and right parietal atrophy with grade 1 subcortical leukoencephalopathy (coronal FLAIR) (B). (C) Family pedigree and genotype data for the *SPG7* variants. Symbols: square, male; circle, female; filled, affected individual; half‐filled, clinically healthy carriers; asterisk, individual sequenced by WES/WGS. Variants found in SPG7 are shown below each symbol. WT: wild‐type. (D) Agarose gel electrophoresis of RT‐PCR products showing an additional fragment (513 bp) in patient II.1. No products were observed in negative RT controls or water controls (blank). A healthy family member (II.2) not carrying the c.286 + 853A>G variant and an unrelated individual were used as healthy controls. (E) Consequence of the c. 286 + 853 A> G variant. WT: structure of wild‐type *SPG7* transcript (exons 1–4). MUT: structure of *SPG7* transcript generated by the c. 286 + 853 A> G variant. The pseudoexon results from a cryptic donor splice site activation upstream from the c.286 + 853A>G mutation. The in‐frame inclusion of 75 nucleotides of intron 2 (blue, pseudoexon ᴪ2b) translates into premature stop codons at positions 1 and 4 downstream. Arrows indicate primers used for RT‐PCR. cDNA Sanger sequencing shows two populations of mRNA, one corresponding to pseudoexon inclusion and the other to exon 3. (F) Western blot of OPA1 and SPG7 proteins for human fibroblasts of individuals II.1 (patient), II.2 (healthy brother) and controls (*n* = 3). Right, quantification of western blot bands. Data represented as mean ± SD

A complete diagnostic workup was undertaken to exclude any acquired cause of spastic paraparesis or gait ataxia. Full blood analysis was normal, including absence of acanthocytosis, and negative antineuronal, onconeuronal, and ganglioside antibodies. Only antinuclear antibodies were positive in 1/640 titers for several analyses, but a thorough rheumatology study was unremarkable, including negative autoantibodies and normal salivary gland biopsy. Oxysterol levels ruled out Niemann Pick disease. CSF analysis was also unremarkable. On MRI, there was bilateral frontotemporal and right parietal atrophy with grade 1 subcortical leukoencephalopathy. No T2 hyperintensities were seen in the dentate nuclei. There was concomitant cerebellar atrophy and no signs of myelopathy (Fig. 1A and 1B). Whole‐body muscle‐MRI showed no myopathic changes, and thoracoabdominopelvic CT scan was negative for any mass suggestive of malignancy. There was no evidence of polyneuropathy or myopathy on nerve conduction studies and electromyography. Genetic analysis for different spinocerebellar ataxias (SCA1, 2, 3, 6, 7), DRPLA and Friedreich ataxia were negative. Furthermore, no mutations were found in the mitochondrial DNA.

The patient's disability has progressed slowly, and at the last visit (May 2019), he is currently able to walk short distances with a walker or cane, requiring a wheelchair for the rest of activities. The Spastic Paraplegia Rating Scale (SPRS) was 15/52 (1,3,2,2,2,2,1,1,1,0,0,0,0), the timed 25‐foot walk test (T25FW) was 15.94 sec, and the 6‐min walk test (6MWT) was 177 meters. No waddling or signs of proximal myopathy were observed. There has been no cognitive or behavioral decline up to this date. He continues to show cerebellar dysarthria. A slight improvement was observed after 1 month of fampridine (Fampyra) (T25FW = 13.73 sec), while other symptomatic treatments failed.

### Molecular studies

WES analysis identified a heterozygous missense variant in *SPG7* (chr16:89623308T> C hg19, NM_003119.3:c.2195T> C, p.Leu732Pro), which was inherited from his father (Fig. 1C). This variant is completely absent from control databases, is highly conserved in evolution (GERP score: 5.59), is predicted to be deleterious by at least three different predictors (PolyPhen‐2, SIFT, MutationTaster), and is located in the conserved peptidase M41 domain, close to other described missense mutations. Based on these data, we classified the variant as “Likely Pathogenic” according to ACMG/AMP guidelines.^10^ As the clinical presentation of this patient was compatible with mutations in *SPG7*, and as we did not find other rare candidate variants in exome data for this patient, we decided to perform WGS on this patient's sample to seek a second variant in *SPG7*, while using better coverage to rule out other possible disease‐causative genes or novel candidates that could underlie our patient's phenotype.

Indeed, while WGS did not detect candidate variants of interest in other HSP or ataxia genes, it revealed a novel heterozygous variant within intron 2 of the *SPG7* gene, which was inherited from the mother (Fig. 1C). This variant (chr16:89577853A> G hg19, NM_003119.3:c.286 + 853A>G) is deep intronic (>100 bp from the splice site), completely absent from control databases, and has not been previously reported as associated with disease. Segregation in two healthy siblings was compatible with pathogenicity of the variant. *In silico* analysis using various splice site predictors suggested the activation of a cryptic donor site 5 bp upstream from this variant (Table S2). Furthermore, SpliceAI, a very novel tool to predict cryptic splice sites based on deep learning of artificial neural networks,^9^ additionally predicted the activation of an acceptor site located 80 bp upstream from the c.286 + 853A>G variant at c.286 + 773. This novel acceptor site should function in conjunction with the previous donor site of exon 2 at position c.286 + 1, leading to the inclusion of a cryptic exon from c.286 + 774 to c.286 + 848. The effect appeared specific for the change c.286 + 853A>G, as no donor or acceptor activation events were predicted by SpliceAI for other nucleotide changes, such as c.286 + 853 A> C or A> T. Thus, we set out to validate the prediction and extracted cDNA from the patient's fibroblasts, a healthy sibling, and an additional control. Gel analysis and Sanger sequencing revealed that a 75 bp in‐frame pseudoexon was retained in the cDNA as a consequence of the c.286 + 853A>G variant, matching the SpliceAI prediction (Fig. 1D). This novel mRNA species contains an immediate, premature stop codon at p.(Gly96Gly*1), which is supposed to induce degradation of the transcript by nonsense mediated decay (NMD) (Fig. 1E). No evidence of transcripts containing this pseudoexon was found in the RNA‐seq data of control fibroblasts from the Genotype‐Tissue Expression project (GTEx, https://gtexportal.org). Ensuing this functional validation, the c.286 + 853A>G variant can be classified as “Pathogenic” according to ACMG/AMP guidelines.^10^ Western blot analyses showed that SPG7 protein was firmly decreased in patient II.1 fibroblasts compared to controls, in concordance with the expected loss‐of‐function effect of the deep intronic variant, while his healthy brother showed a less severe reduction in SPG7 levels, possibly linked to an effect of the p.Leu732Pro variant on protein stability (Fig. 1F). On the other hand, OPA1 protein levels were strongly upregulated in patient II.1, a feature that has also been observed in other SPG7‐mutated patients.^11^ Thus, protein analysis further confirmed the implication of SPG7 variants on our patient's phenotype.

## Discussion

In this work, we present a case in which WGS was indispensable to identify the causative mutations in an HSP patient. Although various studies in the last years have used WGS to study the etiology of HSP, the variants identified in these works were located near or inside coding regions and could very probably have been detected by WES (Table S1). In contrast, our patient harbored a deep intronic variant inducing the inclusion of a pseudoexon and premature stop sites, which could not have been identified with WES alone, underscoring the usefulness of WGS to increase diagnostic yield. Alternatively, transcriptome sequencing could have been used, as it has recently been proven useful for other genetic diseases.^12^, ^13^ Deep intronic mutations are estimated to account for 10% of pathogenic mutations in the general rare genetic disorders population,^9^, ^14^ and could underlie a significant number of undiagnosed HSP/ataxia cases as it has been shown in our case,^15^, ^16^ although it is still underreported.

This case, in which only one coding variant was detected at first, highlights that the existence of dominant variants in *SPG7* should be reexamined. To date, the main argument supporting a dominant inheritance of some variants is the description of four families with patients with two mutations having an affected relative carrying only one mutated allele and confirmed by Sanger sequencing.^17^, ^18^, ^19^ Moreover, at least 10 apparently dominant HSP families have been reported, in which the parents of index patients with two mutations *in trans* were also affected, although their carrier status was not confirmed by Sanger sequencing.^20^ However, a full screening of *SPG7* in heterozygous affected relatives was not undertaken in any of these works. It is tempting to speculate that some heterozygous patients in these families in fact carry a third pathogenic allele that would have eluded identification in a first screening, thus mimicking a dominant or pseudodominance pattern in the first instance. Indeed, three families with a pseudodominant mode of inheritance have already been described, with three different pathogenic alleles segregating in the family.^21^, ^22^ It is worth noting that the p.Ala510Val pathogenic variant is relatively frequent in public control databases (0.25% in ExAC, 0.4% in European population) and was carried in homozygotes by some affected individuals in the three families mentioned. Although the pseudodominance scenario would not reconcile well with the existence of *SPG7*‐mutated families with three affected generations described in literature, we believe that full gene screening in the apparently heterozygous individuals should be required for claiming the existence of dominant mutations in *SPG7* in future cases, including non‐coding regions undetectable by WES. As suggested by this case description, WGS and cDNA/transcriptome sequencing will become key diagnostic tools for uncovering mutations undetectable by WES. In addition, these techniques could also help to assess the pathogenicity of *SPG7* variants classified as variants of unknown significance (VUS), while screening for the presence of additional candidate variants in deep intronic regions. This screening will be crucial to better determine the pathogenicity of *SPG7* variants and improve current clinical management and genetic counseling for these families.

Finally, the reported case is affected by cerebellar ataxia, a frequent feature in *SPG7*‐mutated patients (65–90% patients),^20^, ^22^, ^23^ which was clearly predominant over the spastic paraplegia component, and dysarthria (up to 76% of patients in a recent cohort).^23^ Our patient only harbors one heterozygous loss‐of‐function mutation (the deep intronic cryptic splice variant), which is in line with his predominantly ataxic phenotype, as a recent analysis of 249 patients associated biallelic loss‐of‐function mutations in *SPG7* with stronger spasticity phenotypes.^20^ This patient also had PEO and a high degree of disability, which are less common features in *SPG7*‐mutated patients. PEO is present in ~ 26% of Spanish patients, although frequency doubles in patients not carrying the common p.Ala510Val mutation in homozygosis.^22^, ^24^ Given that *SPG7* is one of the most frequent causes of HSP, and also a relatively frequent cause underlying hard‐to‐diagnose ataxias,^25^ it would be prudent to search for intronic *SPG7* variants in patients with ataxia or spastic paraplegia, especially if they have associated PEO, or are already known to harbor one suspicious heterozygous variant in this gene.

## Author Contribution

AP and CC provided funding to run the study. EV, GFE, ALM, and AP designed and conceptualized the study. EV, AS, GFE, RRM, MZ, LPS, MR, and SF performed the analysis and interpreted the data. EV, GFE, ALM, and AP drafted the manuscript. All authors critically revised the manuscript.

## Conflict of Interests

The authors report no disclosures or conflicts of interest relevant to the manuscript.

## Supporting information


**Table S1**. Previous descriptions of HSP patients sequenced by WGS
**Table S2**. Splice prediction scores obtained for the c.286+853A>G mutation
