# Supplementary material for: A deep intronic splice variant advises reexamination of presumably dominant SPG7 Cases
Source: Ann Clin Transl Neurol. 2019 Dec 18;7(1):105–11. doi: 10.1002/acn3.50967 (PMC6952318; doi:10.1002/acn3.50967)
Supplement: Supplementary file 1 — Table S1. Previous descriptions of HSP patients sequenced by WGS Table S2. Splice prediction scores obtained for the c.286+853A>G mutation [file ACN3-7-105-s001.docx]

Supplemental Table 1: Previous descriptions of HSP patients sequenced by WGS

| **Article** | **Patients** | **Mutations detected** |
| --- | --- | --- |
| Yu *et al*., 2016 (1) | 2 HSP patients (1 family) | ***SPG11***: c.6856C>T (p.Arg2286Ter); c.2316+5G>A; compound heterozygous |
| Kumar *et al*., 2016 (2) | 9 HSP patients (9 different families, 4 diagnosed) | ***DDHD2*** : c.1125+1G>T, homozygous  ***PEX16*** : c.995_997delTCT (p.Phe332del), homozygous  ***CYP2U1***: c.782_785delTCTG (p.Cys262Ter), homozygous  ***GLB1***: c.553-2A>G; c.1325G>A (p.Arg442Gln); compound heterozygous |
| Elsaid *et al*., 2017(3) | 2 HSP patients (1 family) | ***NT5C2***: c.1159+1G>T (p.Gly330_Ser387del), homozygous |
| Darvish *et al*., 2017 (4) | 3 HSP patients (1 family) | ***NT5C2***: c.771+573_814-298del (p.Lys258_Lys271del), 1.9Kb homozygous deletion |
| Kumar *et al*., 2018(5) | 1 patient with Leukodystrophy and Spastic paraplegia  (1 family) | ***PEX16***: c.658G>A (p.Ala220Thr); c.830G>A (p.Arg277Gln); compound heterozygous |
| Jia *et al*., 2018 (6) | 38 patients diagnosed with Multiple Sclerosis. 2 Patients harbored mutations in HSP genes, phenocopying Multiple Sclerosis | ***KIF5A***: c.1082C>T (p.Ala361Val), heterozygous  ***REEP1***: c.*43G>T (3’UTR), heterozygous |
| Kim *et al*., 2019 (7) | 18 HSP patients (18 different families, 7 diagnosed) | ***ABCD1***: c.644T>C (p.Leu215Pro), hemizygous  ***ABCD1***: c.346_348dupGGA (p.Gly116dup), heterozygous  ***ABCD1***: c.1866-10G>A, hemizygous ***CAPN1***: c.1271T>G (p.Met424Arg); c.1943-5_1943del ACCAGG; compound heterozygous  ***CAPN1***: c.614T>G (p.Leu205Arg); c.1142C>T (p.Ala381Val); compound heterozygous  ***NIPA1***: c.316G>A, (p.Gly106Arg), heterozygous  ***PLA2G6***: c.278C>A (p.Pro93His); c.1634A>G (p.Lys545Arg); compound heterozygous |

Supplemental Table 2: Splice prediction scores obtained for the c.286+853A>G position

|  | **WT (A)** | **MUT (G)** |
| --- | --- | --- |
| SpliceAI (8)  [0-1] | No score | 0.80 (novel donor site)  SpliceAI also predicts the activation of a cryptic acceptor site located 79 bp upstream (score: 0.60) |
| NNSplice (9)  [0-1] | No score | 0.87 (novel donor site) |
| NetGene2 (10) [0-1] | No score | 0.44 (novel donor site) |
| FSplice (11) | No score | 9.88 (threshold for 90% of true positive splice sites: 6.099) (novel donor site) |
| Human Splicing Finder (12) | 73.97 | 86.13 (+ 16.44%) (novel donor site) |
| MaxEntScan (13) | 1.18 | 6.91 (+ 685%) (novel donor site) |

**BIBLIOGRAPHY**

1. Yu AC-S, Chan AY-Y, Au WC, Shen Y, Chan TF, Chan H-YE. Whole-genome sequencing of two probands with hereditary spastic paraplegia reveals novel splice-donor region variant and known pathogenic variant in *SPG11*. Mol Case Stud. 2016 Nov;2(6):a001248.

2. Kumar KR, Wali GM, Kamate M, et al. Defining the genetic basis of early onset hereditary spastic paraplegia using whole genome sequencing. Neurogenetics. 2016 Oct 28;17(4):265–70.

3. Elsaid MF, Ibrahim K, Chalhoub N, Elsotouhy A, El Mudehki N, Abdel Aleem A. NT5C2 novel splicing variant expands the phenotypic spectrum of Spastic Paraplegia (SPG45): case report of a new member of thin corpus callosum SPG-Subgroup. BMC Med Genet. 2017 Mar 21;18(1):33.

4. Darvish H, Azcona LJ, Tafakhori A, Ahmadi M, Ahmadifard A, Paisán-Ruiz C. Whole genome sequencing identifies a novel homozygous exon deletion in the NT5C2 gene in a family with intellectual disability and spastic paraplegia. npj Genomic Med. 2017 Dec 1;2(1):20.

5. Kumar KR, Wali G, Davis RL, et al. Expanding the spectrum of PEX16 mutations and novel insights into disease mechanisms. Mol Genet Metab reports. 2018 Sep;16:46–51.

6. Jia X, Madireddy L, Caillier S, et al. Genome sequencing uncovers phenocopies in primary progressive multiple sclerosis. Ann Neurol. 2018 Jul;84(1):51–63.

7. Kim A, Kumar KR, Davis RL, et al. Increased Diagnostic Yield of Spastic Paraplegia with or Without Cerebellar Ataxia Through Whole-Genome Sequencing. The Cerebellum. 2019 May 18;

8. Jaganathan K, Kyriazopoulou Panagiotopoulou S, McRae JF, et al. Predicting Splicing from Primary Sequence with Deep Learning. Cell. 2019 Jan 24;176(3):535-548.e24.

9. REESE MG, EECKMAN FH, KULP D, HAUSSLER D. Improved Splice Site Detection in Genie. J Comput Biol. 1997 Jan;4(3):311–23.

10. Brunak S, Engelbrecht J, Knudsen S. Prediction of human mRNA donor and acceptor sites from the DNA sequence. J Mol Biol. 1991 Jul 5;220(1):49–65.

11. FSPLICE - find splice sites in genomic DNA [Internet]. [cited 2019 Aug 1]. Available from: http://www.softberry.com/berry.phtml?topic=fsplice&group=programs&subgroup=gfind

12. Desmet F-O, Hamroun D, Lalande M, Collod-Béroud G, Claustres M, Béroud C. Human Splicing Finder: an online bioinformatics tool to predict splicing signals. Nucleic Acids Res. 2009 May;37(9):e67.

13. Yeo G, Burge CB. Maximum Entropy Modeling of Short Sequence Motifs with Applications to RNA Splicing Signals. J Comput Biol. 2004 Mar;11(2–3):377–94.
